# Potentially Inappropriate Medication Prescribing Detected by Computer Algorithm among Older Patients: Results from the MAPT Study

**DOI:** 10.3390/pharmacy9040189

**Published:** 2021-11-24

**Authors:** Arnaud Pagès, Laure Rouch, Nadège Costa, Philippe Cestac, Philipe De Souto Barreto, Yves Rolland, Bruno Vellas, Laurent Molinier, Blandine Juillard-Condat, MAPT/DSA Group

**Affiliations:** 1 Department of Pharmacy, Toulouse University Hospital, 31000 Toulouse, France; rouch.l@chu-toulouse.fr (L.R.); cestac.p@chu-toulouse.fr (P.C.); juillard-condat.b@chu-toulouse.fr (B.J.-C.); 2 Institute of Aging, Gérontopôle, INSPIRE Project, Toulouse University Hospital, 31000 Toulouse, France; desouto-barreto.p@chu-toulouse.fr (P.D.S.B.); rolland.y@chu-toulouse.fr (Y.R.); vellas.b@chu-toulouse.fr (B.V.); 3 Center for Epidemiology and Research in POPulation Health (CERPOP), UMR 1295, Inserm, UPS Toulouse III University, 31000 Toulouse, France; costa.n@chu-toulouse.fr (N.C.); molinier.l@chu-toulouse.fr (L.M.); 4 Economic Evaluation Unit, Medical Information Department, Toulouse University Hospital, 31000 Toulouse, France

**Keywords:** potentially inappropriate medication prescribing, medication, elderly, algorithm, database

## Abstract

(1) Background: Some medications may be dangerous for older patients. Potentially inappropriate medication prescribing (PIP) among older patients represents a significant cause of morbidity. The aim of this study was to create an algorithm to detect PIP in a geriatric database (Multidomain Alzheimer Preventive Trial (MAPT) study), and then to assess the algorithm construct validity by comparing the prevalence of PIP and associated factors with literature data. (2) Methods: An algorithm was constructed to detect PIP and was based on different explicit criteria among which the European list of potentially inappropriate medications (EU(7)-PIM), the STOPP and START version 2 tools. For construct validity assessment, logistic mixed-effects model repeated measures analyses were used to identify factors associated with PIP. (3) Results: Prevalence of PIP was 59.0% with the EU(7)-PIM list criteria, 43.2% with the STOPP criteria and 51.3% with the START criteria. Age, polypharmacy, and higher Charlson comorbidity index were associated with PIP. (4) Conclusions: Prevalence of PIP and associated factors are consistent with literature data, supporting the construct validity of our algorithm. This algorithm opens up interesting perspectives both in terms of analysis of very large databases and integration into e-prescribing or pharmaceutical validation software.

## 1. Introduction

Older patients, who often suffer from multiple comorbidities and take multiple medications, are particularly at risk for potentially inappropriate use of medications, including overuse, underuse, and misuse. A recent meta-analysis showed an association between potentially inappropriate medication prescriptions (PIPs) and the risk of adverse effects and hospitalizations in older subjects [1].

Several tools have been developed to make PIPs identification easier, based on explicit or implicit approaches, or a combination of both. The *implicit approach* is based on clinical judgment: the risk/benefit ratio of each medication is analyzed with regard to medical history, comorbidities, laboratory tests, and co-prescribed medications. The Medication Appropriateness Index (MAI) developed by Hanlon et al. is based on this approach, which is characterized by significant inter-observer variability [2]. The *explicit approach* is based on criteria that are derived from expert consensus. These criteria are simple to use and can be applied in the same way in all patients, regardless of individual characteristics. It involves standardized lists of medications to be avoided in older patients [3,4]. A literature review identified 36 validated explicit criteria-based tools for identifying PIPs in older people [5]. Initially, these were simple lists of potentially inappropriate medications (Beers Criteria, and the European List of Potentially Inappropriate Medications for Older People (EU(7)-PIM)) [3,6]. These tools gradually became more complex, in the form of multiple-criteria rules that incorporated clinical and laboratory data [4]. This made them more sensitive but less practical to use [4].

PIP identification tools are used both in routine practice to improve individual patient safety and in research to measure PIPs frequency and give insight into the risk factors associated with PIPs. In this second case, PIPs are detected in large databases, using either lists of potentially inappropriate medications [7] or algorithms that translate multiple criteria rules [8]. The algorithms integrating the clinical, biological and prescription data were first designed to detect potentially inappropriate medications [9,10] and then permitted to detect both prescriptions omissions and potentially inappropriate medications, based on STOPP/START v1 criteria [11]. An algorithm based on several rules (START and STOPP v2 criteria and the Beers Criteria) is announced in the COME-ON study protocol (Collaborative approach to Optimize MEdication use for Older people in Nursing homes) [8,12].

In this context, the first objective of this study was to develop an algorithm to detect both inappropriate medications, major drugs interactions and prescriptions omissions, based on four explicit criteria-based tools (including the EU(7)-PIM list and START and STOPP v2 criteria). The secondary objective was to test the applicability of the algorithm on a large geriatric database and to assess its construct validity by identifying factors that have previously shown a relationship with PIP.

## 2. Materials and Methods

### 2.1. Database

We used the data from the Multidomain Alzheimer Preventive Trial (MAPT) to conduct a longitudinal secondary analysis. The Multidomain Alzheimer Preventive Trial (MAPT) was a 3-year, multicenter (13 memory centers in France and Monaco), randomized trial aimed to test the preventive effect of omega-3 supplementation or multidomain intervention (nutritional and exercise counseling and cognitive training) or both on dementia and follow-up data every 6 months. Participants were aged 70 years or older, and community-dwelling without dementia or any difficulty in basic activities of daily living (ADL) [13] at baseline. The multidomain intervention was not focused on medication review [14]. The trial protocol was approved by the French Ethical Committee located in Toulouse (CPP SOOM II). This specific secondary analysis was approved for ethics and feasibility by the Multidomain Alzheimer Preventive Trial/Data Sharing Alzheimer (MAPT/DSA) Group (Appendix A).

The database included prescription and over-the-counter (OTC) medications, with the medication name, International Non-proprietary Name, Anatomical Therapeutic Chemical (ATC) fifth level class (classification system of active substances according to their target organs and their chemical, pharmacological or therapeutic properties, 5th level is the chemical substance), dosage, and start and end dates. Comorbidities (free text coded) and their date of onset were available in the database. We calculated the Charlson comorbidity index for each patient at baseline [15]. The database also contained other clinical parameters (Fried frailty phenotype [16], systolic and diastolic blood pressure in supine and standing positions, heart rate, geriatric depression scale (GDS) [17], etc.).

### 2.2. Algorithm

We developed our own computerized PIP detection algorithm from various explicit criteria-based tools:

- The European List of Potentially Inappropriate Medications in Older People (EU(7)-PIM list) [3].
- The STOPP and START version 2 criteria (Screening Tool of Older People’s Prescriptions and Screening Tool to Alert to Right Treatment) [4].
- Clinical practice indicators of Alert and Mastering of medication Iatrogenicity (AMI) proposed by the French National Authority for Health (HAS) related to medication prescriptions in older patients [18].
- Market withdrawals by the French National Agency for Medicines and Health Products Safety (ANSM) or the European Medicines Agency (EMA) for safety reasons.
- Contraindications listed in the medications’ Summary of Product Characteristics (SmPC).

As there are many explicit criteria tools available, we chose EU(7)-PIM list, the STOPP and START version 2 criteria because they were the most recent tools in Europe. In addition, they were validated for both inpatients and outpatients and the combination of the two allowed us to detect situations of overuse, misuse and underuse [5]. We added three other tools to comply with the recommendations of French and European health agencies related to medication prescription. We identified market withdrawals manually from the websites of the agencies concerned and identified contraindications by querying a medication database approved by the French National Authority for Health. During this development, we identified contradictions or redundancies between the various explicit criteria-based tools. The choice of the combination of several criteria was guided by the objective of relating as much as possible to the pharmaceutical analysis carried out by a clinical pharmacist who uses all available sources.

A clinical pharmacist with expertise in geriatrics coded explicit criteria. Another pharmacist then checked the entire coding. In case of disagreement on a part of the code, a conciliation was performed and a decision was made by consensus between the two pharmacists. The programming of the computer algorithm was performed with SAS© 9.3 software (SAS Institute Inc., Cary, NC, USA). 

Some explicit criteria required methodological choices for coding. For the STOPP A1 criterion “Any medication prescribed without an evidence-based clinical indication”, we only coded two sub-criteria proposed by a panel of Belgian, Canadian, French and Swiss experts (1: aspirin and statin in primary cardiovascular prevention, 2: proton pump inhibitor without recent oesogastric damage) [19]. For the STOPP B6 criterion “Loop diuretic as first-line treatment for hypertension”, we considered hypertensive patients with a loop diuretic, without heart failure, liver failure, nephrotic or renal syndrome and without any other concomitant antihypertensive medication. For the START G1 and G2 criteria related respectively to alpha-1 receptor blocker and alpha reductase inhibitor with symptomatic prostatism, we could not consider "where prostatectomy is not necessary", we only considered whether prostatectomy had been performed. In addition, dosage information was used to distinguish medicines indications (e.g., antiplatelet agents vs. non-steroidal anti-inflammatory medications) or to clarify the coding of certain criteria.

Some criteria were not considered relevant for coding. The STOPP K1 (benzodiazepines), K2 (neuroleptic drugs), and K4 (hypnotic Z-drugs) criteria were considered too broad to be clinically relevant to assessors, and appeared to be partially redundant with other more specific criteria (e.g., STOPP D3, D5, or G4 criteria). The START C3 criterion “acetylcholinesterase inhibitor for mild/moderate Alzheimer’s dementia or Lewy Body dementia” was not taken into account since these medications are no longer recommended in France. Six clinical practice indicators AMI were considered to be prescription monitoring criteria more than PIP detection criteria, and therefore, were not coded. The other uncoded criteria were related to the unavailability of certain variables in the database.

Then, the computer algorithm was used on the database combining the different datasets (medication prescription, comorbidities, clinical data (e.g., blood pressure, assessment of frailty)) of the MAPT study. The computer algorithm generated a number of medication-related potential noncompliances (MRNC) by prescription at each follow-up time. We took into account strict duplicates between tools when counting the number of MRNC. A medication prescription was considered potentially inappropriate (PIP) if it included at least one MRNC. The PIP variable was thus coded in a binary manner for each patient and each follow-up time.

### 2.3. Statistical Analysis

As we made the hypothesis that data were missing at random (MAR), the missing data were imputed by Multiple Imputation by Chained Equations (MICE) [20] if it exceeded 1%. The baseline numbers and percentages for the categorical variables were presented; comparisons were made using the χ² test (or Fisher’s exact test (for expected values < 5)). To verify the construct validity of the computer algorithm, we performed a multivariate analysis to assess the association between patient characteristics (age, gender, number of medications prescribed, Charlson comorbidity index, education level, frailty) and PIP. These patient characteristics were chosen because they were frequently associated with PIP in other studies [21,22]. We also introduced MAPT intervention groups and follow-up time (categorical variable) in the model. The age groups were defined according to two thresholds: 75 years and 80 years [18]. We categorized the number of medications prescribed into three categories: non-polypharmacy (0–4 medications), polypharmacy (5–9 medications), and hyperpolypharmacy (10 or more medications) [23]. Polypharmacy, frailty and PIP were considered as time-varying variables. We conducted a multivariate analysis using a mixed logistic regression model with a random effect at the level of patients to consider the correlation of observations for a single individual (repeated measures) and fixed effects for other variables [24]. The results were presented as an odds ratio for each variable included in the analysis. All hypothesis testing was performed using a 5% significance level (*p* values and corresponding 95% confidence intervals for each odds ratio). The model programming and statistical analyses were done using the software SAS© 9.3 (SAS Institute Inc., Cary, NC, USA).

## 3. Results

**Figure 1:**
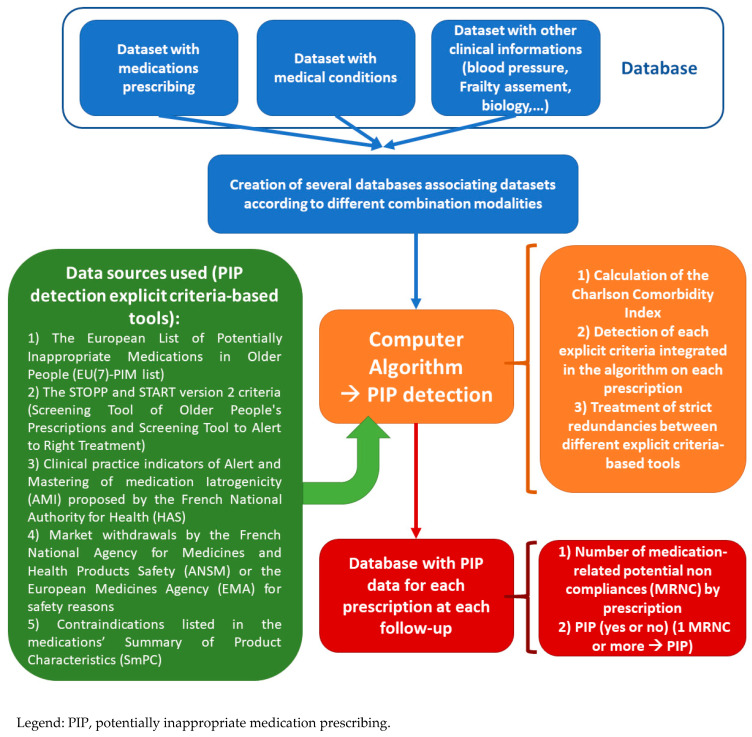
Solution procedure flow chart of the Computer Algorithm.

The solution procedure flow chart of the computerized PIP detection algorithm is presented in Figure 1. The coded criteria and information used, and the strict redundancies, are explained in Table S1 in the Supplementary Material. We were able to code 47 of 80 STOPP criteria, 24 of 34 START criteria, five of 11 AMI criteria, and the entire European list EU(7)-PIM (282 medications or medication classes), 21 marketing authorization (MA) withdrawals and 7457 contraindications. Overall, the algorithm analyzed 9643 medication prescriptions from 1525 patients.

**Figure 2:**
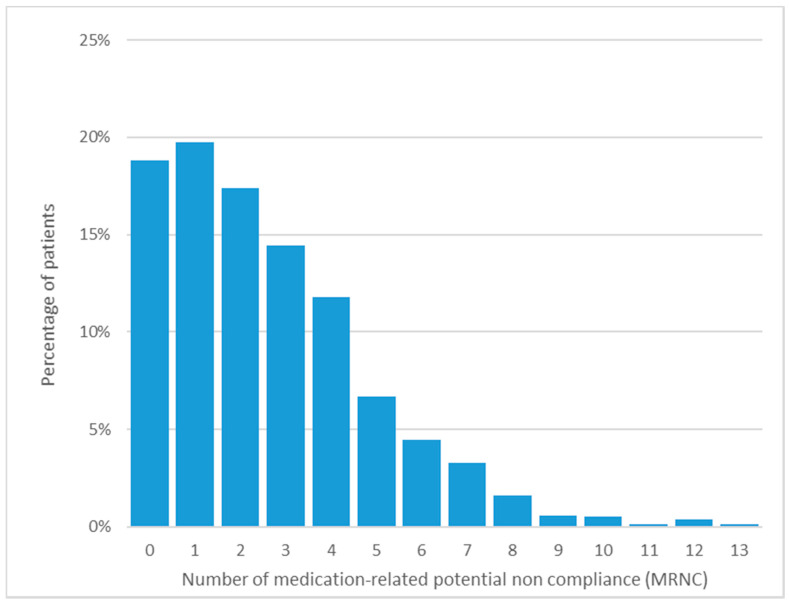
Percentage of medication-related potential noncompliance occurrences at baseline.

Baseline characteristics are shown in Table 1, and the percentage of patients according to the number of MRNCs is presented in Figure 2. At baseline, frailty assessment and education level were missing in 5% and 2% of cases respectively.

At baseline, before accounting for duplicates between the tools, out of 1525 patients, we found at least one PIP in 900 patients (59.0%) with the EU(7)-PIM list criteria, 659 patients (43.2%) with the STOPP criteria, 783 patients (51.3%) with the START criteria, 212 patients (13.9%) with the French AMI criteria; for 13 patients (0.9%) a contraindication due to possible drug interaction was detected in the prescription (Table 2).

Regarding the construct validity assessment of the computer algorithm, we found that age, polypharmacy, and higher Charlson comorbidity index were associated with PIP in the multivariate analysis (Table 3).

## 4. Discussion and Conclusions

We incorporated in the computer algorithm the entire EU(7)-PIM list, 59% (47/80) of STOPP criteria and 70% (24/34) of START criteria. Prevalence of PIP was 59.0% with EU(7)-PIM list criteria, 43.2% with STOPP criteria and 51.3% with START criteria. In the multivariate analysis, we found older age, polypharmacy, and a high comorbidity index to be associated factors with PIPs. Conversely, neither the intervention group of the MAPT study nor the level of education was associated with PIPs.

It should be emphasized that PIP detection tools were first designed for use by clinicians, who have complete clinical data as well as the patient’s care history. Coding of these tools within algorithms, followed by application of algorithms to research databases, both lead to several limits. 

Firstly, the main encoding difficulties arise from the fact that some criteria are not precisely defined and leave a margin of interpretation; others need clarification to avoid false positives [8]. Secondly, PIP detection on large research databases may be limited by the unavailability of clinical or biological data. In fact, 85% of the STOPP criteria and 97% of the START criteria require other information in addition to medication prescription data (laboratory and clinical parameters, or medication histories) [8]. In our study, the combination of our coding and data available in the database allowed us to take into account 59% of STOPP criteria and 70% of START criteria, which is literature data: respectively 40% and 59% of STOPP and START v1 criteria were applied on a database of 3454 Irish adults aged ≥65 years [25]; Nauta et al. were able to apply 63% of the STOPP v1 criteria and 69% of the START v1 criteria on a Dutch primary care database, including 1187 patients aged ≥65 years [11]. Regarding validation of computer algorithms applied to research databases, Nauta et al. stress that there is no gold standard for evaluating the appropriateness of a prescription, and choose to estimate the validity of how its algorithm is built by comparing the characteristics of the PIPs detected with those found in published studies [11]. Two characteristics can be compared: prevalence of PIP, and factors associated with PIP.

First, it must be emphasized that PIP prevalence assessed on a large database using algorithms should not be considered as a reliable indicator of the clinical relevance of prescriptions: in our study, potential inappropriateness is considered as a dichotomized variable, and results are only intended to validate the construction of the algorithm. The comparability of the PIP prevalence results is mostly limited by criteria actually coded during detection by computer algorithm, and less importantly by differences in patients’ characteristics (outpatients or inpatients, age), and the PIP detection methods used (implicit and/or explicit approach) [21]. In our study on 1525 outpatients over age 70, the prevalence of patients whose prescription contains at least one STOPP alert is 43.2% (47 criteria evaluated), one START alert 51.3% (24 criteria evaluated), one EU(7)-PIM list alert 59.0%, respectively.

A study performed on 38,229 outpatients over age 65, with START and STOPP v2 criteria (45 STOPP criteria evaluated) showed that the prevalence of PIP was 45.3% in 2012 and 51.0% in 2015 [26]. One other study estimated that—out of 503 outpatients—56% had a PIP according to STOPP v2 criteria (46/80 coded criteria) and 67% PPO according to START v2 criteria (13/34 coded criteria) [27]. The higher prevalence relative to our study can be explained by an older population (>80 years). Regarding PIP detection based on the EU(7)-PIM list, one Lithuanian retrospective, population-based study (n = 431,625) of patients over age 65, estimated a prevalence of 57.2%, close to that found in our study (59.0%) [7].

Regarding factors associated with PIP, we found in the multivariate analysis that age, polypharmacy, and higher Charlson comorbidity index are associated with PIP. The finding of the association between PIP and age, polypharmacy, and morbidity established in multivariate analyses is widely consistent with other published studies, that enforce the construct validity of the computer algorithm. According to the review by Tommelein et al., all of the publications analyzed (27/27) show an association between PIP and polypharmacy, more than half (6/10) between PIP and comorbidities, and nearly half (12/25) between PIP and advanced age [21]. This is linked to the fact that age and comorbidity score increase polypharmacy, and therefore, the likelihood of having an inappropriate medication. Likewise, the number of comorbidities also increases with age. Regarding other factors identified in our study, Gallagher et al., also show a correlation between potential prescribing omissions and the Charlson comorbidity index [28]. Muhlack et al. show an association between PIPs and frailty, but only for Beers Criteria in patients with dementia [22].

Our study has strengths. First, we designed a computer algorithm to detect PIPs, based on four explicit criteria-based tools and also major drugs interactions, whereas previous studies only integrated one or two explicit criteria-based tools. As the database included over-the-counter (OTC) medications, we can evaluated the appropriateness of using OTC because the EU(7)-PIM list contains OTC medications. Clinical and biological data available in our database allowed us to encode the majority of START and STOPP v2 criteria. Then, we tested the applicability of this computer algorithm on a large longitudinal geriatric database. Several factors support the construct validity of our algorithm. First, the coding of criteria was defined by consensus between pharmacists and double-checked. Then, we found that factors associated with PIPs in our study were consistent with literature data. The main limitation of this study is that the PIP detection computer algorithm was not validated by comparison with human pharmaceutical analysis. Yet such a manual screening of the prescriptions raises two difficulties: firstly, it should only be based on the explicit criteria selected in the study, and secondly, it may concern only a sample of the 9643 prescriptions database.

Future research may also compare PIP detection using an algorithm, with PIP detection based on explicit and implicit approaches conducted by professionals. This comparison would provide information on the performance of the algorithm, and it would help identify the respective contributions of algorithms and humans in analyzing medication prescriptions in older subjects. This information would also allow us to improve the algorithm’s computer code to make it better reflect clinical practice. 

The explicit criteria-based tools used to develop the computer algorithm were adapted for all elderly people. However, our population was not representative of the entire elderly population since the MAPT study included only community-dwelling patients aged 70 years or older, without dementia or any difficulty in basic activities of daily living (ADL) at baseline, and particularly well educated [14]. It could also be interesting to apply our computer algorithm on databases of dependent or dementia patients in nursing homes.

This computerized PIP detection algorithm was created from several data sources. It opens up some interesting perspectives, both in terms of analyzing very large databases and for its integration into e-prescription or pharmaceutical validation software.

## Figures and Tables

**Table 1:** Baseline characteristics of patients with and without a potentially inappropriate medication prescribing (PIP).

Baseline Characteristics	Appropriateness Medication Prescribing		Total (n = 1525)	*p*-Value
	without PIP (n = 287)	with PIP (n = 1238)		
**Gender, n (%)**				
Female	187 (65%)	791 (64%)	978 (64%)	0.688
Male	100 (35%)	447 (36%)	547 (36%)	
**Age (Years), n (%)**				
Age ≤ 74 years	182 (63%)	581 (47%)	763 (50%)	<0.001
75 years ≤ Age ≤ 79 years	74 (26%)	415 (33%)	489 (32%)	
Age ≥ 80 years	31 (11%)	242 (20%)	273 (18%)	
Education, n (%) *				
No diploma or primary school certificate	59 (21%)	275 (23%)	334 (22%)	0.291
Secondary education	95 (34%)	416 (34%)	511 (34%)	
High school diploma	51 (18%)	167 (14%)	218 (15%)	
University level	77 (27%)	361 (29%)	438 (29%)	
**Intervention Group, n (%)**				
Multidomain plus polyunsaturated fatty acids	68 (24%)	306 (25%)	374 (24%)	0.226
Polyunsaturated fatty acids	60 (21%)	321 (26%)	381 (25%)	
Multidomain plus placebo	83 (29%)	307 (25%)	390 (26%)	
Placebo	76 (26%)	304 (24%)	380 (25%)	
**Number of Medications Prescribed**				
Number of medications prescribed ≤ 4	255 (89%)	557 (45%)	812 (53%)	<0.001
5 ≤ Number of medications prescribed ≤ 9 (polypharmacy)	32 (11%)	557 (45%)	589 (39%)	
Number of medications prescribed ≥ 10 (hyperpolypharmacy)	0 (0%)	124 (10%)	124 (8%)	
**Charlson Comorbidity Index, n (%)**				
0 point	269 (94%)	915 (74%)	1184 (78%)	<0.001
1 point	16 (5%)	232 (19%)	248 (16%)	
≥2 points	2 (1%)	91 (7%)	93 (6%)	
Frailty, n (%) **				
Robust patients	187 (69%)	612 (52%)	799 (55%)	<0.001
Prefrail patients	79 (29%)	524 (45%)	603 (42%)	
Frail patients	5 (2%)	38 (3%)	43 (3%)	
**Instrumental Activities of Daily Living, n (%) ^**				
IADL = 8 (no deficit on instrumental activities)	277 (97%)	1181 (95%)	1458 (96%)	0.192
IADL > 8 (deficit on instrumental activities)	8 (3%)	56 (5%)	64 (4%)	
**Medical Conditions, n (%)**				
Hypertension	157 (55%)	857 (69%)	1014 (66%)	<0.001
Myocardial infarction	0 (0%)	88 (7%)	88 (6%)	<0.001
Heart failure	2 (1%)	65 (5%)	67 (4%)	<0.001
Peripheral vascular disease	3 (1%)	43 (3%)	46 (3%)	0.030
Cerebrovascular accident or transient ischemic attack	1 (0%)	41 (3%)	42 (3%)	0.006
Chronic obstructive pulmonary disease	1 (0%)	41 (3%)	42 (3%)	0.006
Peptic ulcer disease	7 (2%)	58 (5%)	65 (4%)	0.090
Diabetes mellitus #	0 (0%)	13 (1%)	13 (1%)	0.145
Severe chronic kidney disease #	1 (0%)	7 (1%)	8 (1%)	0.999
Cancer #	0 (0%)	9 (1%)	9 (1%)	0.223

Legend: PIP, potentially inappropriate medication prescribing, * n = 1501, ** n = 1445, ^ n = 1522, # Fisher’s exact test.

**Table 2:** Percentage of potentially inappropriate medication prescribing (PIP) criteria occurrence at baseline.

PIP Criteria-Based Tools	Baseline Occurrence, n (%)
• European List of Potentially Inappropriate Medications in Older People (EU(7)-PIM list)	900 (59%)
• Screening Tool to Alert to Right Treatment (START) version 2	783 (51%)
• Screening Tool of Older Persons’ Prescriptions (STOPP) version 2	659 (43%)
• Clinical practice indicators of Alert and Mastering of medication Iatrogenicity (AMI) proposed by the French National Authority for Health (HAS) related to medication prescriptions in older subjects	212 (14%)
• Market withdrawals by the French National Agency for Medicines and Health Products Safety (ANSM) or the European Medicines Agency (EMA) for safety reasons	79 (5%)
• Contraindications listed in the medications’ Summary of Product Characteristics (SmPC)	13 (1%)

Legend: PIP = potentially inappropriate medication prescribing.

**Table 3:** Factors associated with variation over time of potentially inappropriate medication prescribing (PIP).

Parameters	Adjusted * Odds Ratio [95CI]	*p*-Value
**Gender**		
Female	Ref.	0.601
Male	0.82 [0.38; 1.74]	
**Age (Years)**		
Age ≤ 74 years	Ref.	0.003
75 years ≤ Age ≤ 79 years	2.75 [1.24; 6.09]	
Age ≥ 80 years	5.36 [1.86; 15.44]	
**Education**		
No diploma or primary school certificate	Ref.	0.590
Secondary education	1.45 [0.61; 3.47]	
High school diploma	0.87 [0.30; 2.51]	
University level	1.57 [0.64; 3.86]	
**Intervention Group**		
Placebo	Ref.	0.308
Multidomain plus polyunsaturated fatty acids	1.25 [0.47; 3.34]	
Polyunsaturated fatty acids	2.48 [0.92; 6.66]	
Multidomain plus placebo	1.20 [0.46; 3.12]	
**Number of Drugs Prescribed**		
Number of drugs prescribed ≤ 4	Ref.	<0.001
5 ≤ Number of drugs prescribed ≤ 9 (polypharmacy)	26.64 [12.29; 57.73]	
Number of drugs prescribed ≥ 10 (hyperpolypharmacy)	662.16 [76.85; 5705.28]	
**Charlson Comorbidity Index (Baseline)**		
0 point	Ref.	<0.001
1 point	10.89 [3.17; 37.35]	
≥2 points	21.93 [1.48; 324.44]	
**Frailty**		
Robust patients	Ref.	0.151
Prefrail patients	1.33 [0.96; 1.84]	
Frail patients	1.95 [0.69; 5.52]	

Legend: PIP = potentially inappropriate medication prescribing; 95CI = 95% confidence interval, * also adjusted on follow-up time (categorical variable: each 6-month follow-up visit).

## Data Availability

The MAPT data are available upon request, following validation of a research proposal, from Multidomain Alzheimer Preventive Trial/Data Sharing Alzheimer (MAPT/DSA) Group (nicola.coley@inserm.fr or guyonnet.s@chu-toulouse.fr).

## Supplementary Materials

The following are available online at https://www.mdpi.com/article/10.3390/pharmacy9040189/s1, Table S1: Details about the potentially inappropriate medication prescribing detection algorithm

## Appendix A. MAPT/DSA Group

### Appendix A.1. MAPT Study Group

***Principal investigator*:** Bruno Vellas (Toulouse); *Coordinator*: Sophie Guyonnet; *Project leader*: Isabelle Carrié; *CRA*: Lauréane Brigitte; *Investigators*: Catherine Faisant, Françoise Lala, Julien Delrieu, Hélène Villars; *Psychologists*: Emeline Combrouze, Carole Badufle, Audrey Zueras; *Methodology, statistical analysis and data management*: Sandrine Andrieu, Christelle Cantet, Christophe Morin; *Multidomain group*: Gabor Abellan Van Kan, Charlotte Dupuy, Yves Rolland (physical and nutritional components), Céline Caillaud, Pierre-Jean Ousset (cognitive component), Françoise Lala (preventive consultation). The cognitive component was designed in collaboration with Sherry Willis from the University of Seattle and Sylvie Belleville, Brigitte Gilbert and Francine Fontaine from the University of Montreal.

***Co-Investigators in associated centers*:** Jean-François Dartigues, Isabelle Marcet, Fleur Delva, Alexandra Foubert, Sandrine Cerda (Bordeaux); Marie-Noëlle-Cuffi, Corinne Costes (Castres); Olivier Rouaud, Patrick Manckoundia, Valérie Quipourt, Sophie Marilier, Evelyne Franon (Dijon); Lawrence Bories, Marie-Laure Pader, Marie-France Basset, Bruno Lapoujade, Valérie Faure, Michael Li Yung Tong, Christine Malick-Loiseau, Evelyne Cazaban-Campistron (Foix); Françoise Desclaux, Colette Blatge (Lavaur); Thierry Dantoine, Cécile Laubarie-Mouret, Isabelle Saulnier, Jean-Pierre Clément, Marie-Agnès Picat, Laurence Bernard-Bourzeix, Stéphanie Willebois, Iléana Désormais, Noëlle Cardinaud (Limoges); Marc Bonnefoy, Pierre Livet, Pascale Rebaudet, Claire Gédéon, Catherine Burdet, Flavien Terracol (Lyon), Alain Pesce, Stéphanie Roth, Sylvie Chaillou, Sandrine Louchart (Monaco); Kristel Sudres, Nicolas Lebrun, Nadège Barro-Belaygues (Montauban); Jacques Touchon, Karim Bennys, Audrey Gabelle, Aurélia Romano, Lynda Touati, Cécilia Marelli, Cécile Pays (Montpellier); Philippe Robert, Franck Le Duff, Claire Gervais, Sébastien Gonfrier (Nice); Yannick Gasnier and Serge Bordes, Danièle Begorre, Christian Carpuat, Khaled Khales, Jean-François Lefebvre, Samira Misbah El Idrissi, Pierre Skolil, Jean-Pierre Salles (Tarbes).

***MRI group*:** Carole Dufouil (Bordeaux), Stéphane Lehéricy, Marie Chupin, Jean-François Mangin, Ali Bouhayia (Paris); Michèle Allard (Bordeaux); Frédéric Ricolfi (Dijon); Dominique Dubois (Foix); Marie Paule Bonceour Martel (Limoges); François Cotton (Lyon); Alain Bonafé (Montpellier); Stéphane Chanalet (Nice); Françoise Hugon (Tarbes); Fabrice Bonneville, Christophe Cognard, François Chollet (Toulouse).

***PET scans group*:** Pierre Payoux, Thierry Voisin, Julien Delrieu, Sophie Peiffer, Anne Hitzel, (Toulouse); Michèle Allard (Bordeaux); Michel Zanca (Montpellier); Jacques Monteil (Limoges); Jacques Darcourt (Nice).

***Medical economics group*:** Laurent Molinier, Hélène Derumeaux, Nadège Costa (Toulouse).

***Biological sample collection*:** Bertrand Perret, Claire Vinel, Sylvie Caspar-Bauguil (Toulouse).

***Safety management*:** Pascale Olivier-Abbal

### Appendix A.2. DSA Group

***DSA Group*:** Sandrine Andrieu, Christelle Cantet, Nicola Coley
