# Supplementary material for: Potentially Inappropriate Medication Prescribing Detected by Computer Algorithm among Older Patients: Results from the MAPT Study
Source: Pharmacy (Basel). 2021 Nov 24;9(4):189. doi: 10.3390/pharmacy9040189 (PMC8628967; doi:10.3390/pharmacy9040189)
Supplement: Supplementary file 1 [file pharmacy-09-00189-s001.zip › pharmacy-1360825-supplementary.pdf]

# Supplementary Material

**Table S1: Details about the potentially inappropriate medication prescribing detection algorithm**

| Criteria | Explanations | Programmed criteria (Done/Not Done) | Reasons for nonprogramming | Medication information needed (ATC 5) (Yes/No) | Dosage information needed (Yes/No) | Duration information needed (Yes/No) | Route information needed (Yes/No) | Association of drugs information needed (Yes/No) | Comorbidity information needed (text) (Yes/No) | Association of comorbidities information needed (Yes/No) | Others clinical measures (Yes/No) | Others clinical measures (details) | Strict redundancy between criteria | Strict redundancy between criteria |
|---|---|---|---|---|---|---|---|---|---|---|---|---|---|---|
| EU(7)-PIM list | European List of potentially inappropriate medications | Done | - | Yes | Yes | Yes | Yes | No | No | - | - | - | Yes, see other criteria | - |
| Withdrawal | Market withdrawals by the French National Agency of Medicines and Health Products Safety (ANSM) or European Medicine Agency (EMA) due to safety concerns | Done | - | Yes | No | No | No | No | No | - | - | - | Yes with EU-PIM | Pioglitazone (A10BG03), Strontium ranelate (M05BX03), Meprobamate (N05BC01, and N05BC51), Acepromazine (N05CX) |
| CI | Contraindications | Done | - | Yes | Yes | No | No | Yes | No | - | - | - | No | - |
| AMI no. 1 | AMI no. 1: Neuroleptics in patients with Alzheimer disease | Done | - | Yes | No | No | No | No | Yes | No | No | - | No | - |
| AMI no. 2 | AMI no. 2: Coprescription of 2 psychotropic drugs in the elderly | Done | - | Yes | No | No | No | Yes | No | - | - | - | No | - |
| AMI no. 3 | AMI no. 3: Long-acting benzodiazepine in the elderly | Done | - | Yes | No | No | No | No | No | - | - | - | Yes with EU-PIM | Numerous Long-acting benzodiazepine s |
| AMI no. 4 | AMI no. 4: Date of initiation of treatment known for all psychotropic drugs | Not Done | Prescription monitoring criteria not suited to a clinical study | - | - | - | - | - | - | - | - | - | - | - |
| AMI no. 5 | AMI no. 5: Search falls every 3 months if psychotropic | Not Done | Prescription monitoring criteria not suited to a clinical study | - | - | - | - | - | - | - | - | - | - | - |
| AMI no. 6 | AMI no. 6: Coprescription of 2 or more diuretics in the elderly | Done | - | Yes | No | No | No | Yes | No | - | - | - | No | - |
| AMI no. 7 | AMI no. 7: Coprescription of 4 or more antihypertensive drugs in the elderly | Done | - | Yes | No | No | No | Yes | No | - | - | - | No | - |
| AMI no. 8 | AMI no. 8: Electrolyte monitoring if increased risk associated with a "loop diuretic + thiazide diuretic" or "antialdosterone + renin angiotensin system inhibitor" | Not Done | Prescription monitoring criteria not suited to a clinical study | - | - | - | - | - | - | - | - | - | - | - |
| AMI no. 9 | AMI no. 9: Weight monitoring if diuretics prescribed | Not Done | Prescription monitoring criteria not suited to a clinical study | - | - | - | - | - | - | - | - | - | - | - |
| AMI no. 10 | AMI no. 10: Search orthostatic hypotension every 6 months if antihypertensive | Not Done | Prescription monitoring criteria not suited to a clinical study | - | - | - | - | - | - | - | - | - | - | - |
| AMI no. 11 | AMI no. 11: INR control if a VKA is prescribed and antibiotics or antifungals started | Not Done | Prescription monitoring criteria not suited to a clinical study | - | - | - | - | - | - | - | - | - | - | - |

| Criteria | Explanations | Programmed criteria (Done/Not Done) | Reasons for nonprogramming | Medication information needed (ATC 5) (Yes/No) | Dosage information needed (Yes/No) | Duration information needed (Yes/No) | Route information needed (Yes/No) | Association of drugs information needed (Yes/No) | Comorbidity information needed (text) (Yes/No) | Association of comorbidities information needed (Yes/No) | Others clinical measures (Yes/No) | Others clinical measures (details) | Strict redundancy between criteria | Strict redundancy between criteria |
|---|---|---|---|---|---|---|---|---|---|---|---|---|---|---|
| STOPP A1 | STOPP A1. Any drug prescribed without an evidence-based clinical indication. | Partially done | Only 2 sub-criteria: aspirin and statin in primary cardiovascular prevention, proton pump inhibitor without recent oesogastric damage. | Yes | No | No | No | No | Yes | No | No | - | No | - |
| STOPP A2 | STOPP A2. Any drug prescribed beyond the recommended duration, where treatment duration is well defined. | Not Done | Lack of comprehensive clinical information to determine the recommended duration of treatment | - | - | - | - | - | - | - | - | - | - | - |
| STOPP A3 | STOPP A3. Any duplicate drug class prescription e.g. two concurrent NSAIDs, SSRIs, loop diuretics, ACE inhibitors, anticoagulants (optimisation of monotherapy within a single drug class should be observed prior to considering a new agent). | Done | - | Yes | No | No | No | No | No | - | - | - | No | - |
| STOPP B1 | STOPP B1. Digoxin for heart failure with normal systolic ventricular function (no clear evidence of benefit) | Not Done | No information on the ventricular ejection fraction in the database | - | - | - | - | - | - | - | - | - | - | - |
| STOPP B2 | STOPP B2. Verapamil or diltiazem with NYHA Class III or IV heart failure (may worsen heart failure). | Done | - | Yes | No | No | No | No | Yes | No | No | - | No | - |
| STOPP B3 | STOPP B3. Beta-blocker in combination with verapamil or diltiazem (risk of heart block). | Done | - | Yes | No | No | No | Yes | No | - | - | - | No | - |
| STOPP B4 | STOPP B4. Beta blocker with bradycardia (< 50/min), type II heart block or complete heart block (risk of complete heart block, asystole). | Done | - | Yes | No | No | No | No | Yes | No | Yes | Heart rate | No | - |
| STOPP B5 | STOPP B5. Amiodarone as first-line antiarrhythmic therapy in supraventricular tachyarrhythmias (higher risk of side-effects than beta-blockers, digoxin, verapamil or diltiazem) | Not Done | No information about "first-line treatment" in the database | - | - | - | - | - | - | - | - | - | - | - |
| STOPP B6 | STOPP B6. Loop diuretic as first-line treatment for hypertension (safer, more effective alternatives available). | Done | - | Yes | No | No | No | No | Yes | No | No | - | No | - |
| STOPP B7 | STOPP B7. Loop diuretic for dependent ankle oedema without clinical, biochemical evidence or radiological evidence of heart failure, liver failure, nephrotic syndrome or renal failure (leg elevation and /or compression hosiery usually more appropriate). | Done | - | Yes | No | No | No | No | Yes | No | No | - | No | - |

| Criteria | Explanations | Programmed criteria (Done/Not Done) | Reasons for nonprogramming | Medication information needed (ATC 5) (Yes/No) | Dosage information needed (Yes/No) | Duration information needed (Yes/No) | Route information needed (Yes/No) | Association of drugs information needed (Yes/No) | Comorbidity information needed (text) (Yes/No) | Association of comorbidities information needed (Yes/No) | Others clinical measures (Yes/No) | Others clinical measures (details) | Strict redundancy between criteria | Strict redundancy between criteria |
|---|---|---|---|---|---|---|---|---|---|---|---|---|---|---|
| STOPP B8 | STOPP B8. Thiazide diuretic with current significant hypokalaemia (i.e. serum K+ < 3.0 mmol/l), hyponatraemia (i.e. serum Na+ < 130 mmol/l) hypercalcaemia (i.e. corrected serum calcium > 2.65 mmol/l) or with a history of gout (hypokalaemia, hyponatraemia, hypercalcaemia and gout can be precipitated by thiazide diuretic) | Done | - | Yes | No | No | No | No | Yes | No | No | - | No | - |
| STOPP B9 | STOPP B9. Loop diuretic for treatment of hypertension with concurrent urinary incontinence (may exacerbate incontinence). | Done | - | Yes | No | No | No | No | Yes | Yes | No | - | No | - |
| STOPP B10 | STOPP B10. Centrally-acting antihypertensives (e.g. methyldopa, clonidine, moxonidine, rilmenidine, guanfacine), unless clear intolerance of, or lack of efficacy with, other classes of antihypertensives (centrally-active antihypertensives are generally less well tolerated by older people than younger people) | Not Done | No information about "clear intolerance of, or lack of efficacy with, other classes of antihypertensives" in the database | - | - | - | - | - | - | - | - | - | - | - |
| STOPP B11 | STOPP B11. ACE inhibitors or Angiotensin Receptor Blockers in patients with hyperkalaemia. | Done | - | Yes | No | No | No | No | Yes | No | No | - | No | - |
| STOPP B12 | STOPP B12. Aldosterone antagonists (e.g. spironolactone, eplerenone) with concurrent potassium-conserving drugs (e.g. ACEI's, ARB's, amiloride, triamterene) without monitoring of serum potassium (risk of dangerous hyperkalaemia i.e. > 6.0 mmol/l – serum K should be monitored regularly, i.e. at least every 6 months). | Not Done | Prescription monitoring criteria not suited to a clinical study and no information about the frequency of serum potassium monitoring in the database | - | - | - | - | - | - | - | - | - | - | - |
| STOPP B13 | STOPP B13. Phosphodiesterase type-5 inhibitors (e.g. sildenafil, tadalafil, vardenafil) in severe heart failure characterised by hypotension i.e. systolic BP < 90 mmHg, or concurrent nitrate therapy for angina (risk of cardiovascular collapse) | Done | - | Yes | No | No | No | Yes | Yes | No | Yes | Systolic blood pressure | No | - |
| STOPP C1 | STOPP C1. Long-term aspirin at doses greater than 160mg per day (increased risk of bleeding, no evidence for increased efficacy). | Done | - | Yes | Yes | Yes | No | No | No | - | - | - | No | - |
| STOPP C2 | STOPP C2. Aspirin with a past history of peptic ulcer disease without concomitant PPI (risk of recurrent peptic ulcer ). | Done | - | Yes | No | No | No | No | Yes | No | No | - | No | - |

| Criteria | Explanations | Programmed criteria (Done/Not Done) | Reasons for nonprogramming | Medication information needed (ATC 5) (Yes/No) | Dosage information needed (Yes/No) | Duration information needed (Yes/No) | Route information needed (Yes/No) | Association of drugs information needed (Yes/No) | Comorbidity information needed (text) (Yes/No) | Association of comorbidities information needed (Yes/No) | Others clinical measures (Yes/No) | Others clinical measures (details) | Strict redundancy between criteria | Strict redundancy between criteria |
|---|---|---|---|---|---|---|---|---|---|---|---|---|---|---|
| STOPP C3 | STOPP C3. Aspirin, clopidogrel, dipyridamole, vitamin K antagonists, direct thrombin inhibitors or factor Xa inhibitors with concurrent significant bleeding risk, i.e. uncontrolled severe hypertension, bleeding diathesis, recent non-trivial spontaneous bleeding) (high risk of bleeding). | Not Done | No information about "recent nontrivial spontaneous bleeding" in the database | - | - | - | - | - | - | - | - | - | - | - |
| STOPP C4 | STOPP C4. Aspirin plus clopidogrel as secondary stroke prevention, unless the patient has a coronary stent(s) inserted in the previous 12 months or concurrent acute coronary syndrome or has a high grade symptomatic carotid arterial stenosis (no evidence of added benefit over clopidogrel monotherapy) | Not Done | No information about "coronary stent insertion date" in the database | - | - | - | - | - | - | - | - | - | - | - |
| STOPP C5 | STOPP C5. Aspirin in combination with vitamin K antagonist, direct thrombin inhibitor or factor Xa inhibitors in patients with chronic atrial fibrillation (no added benefit from aspirin) | Done | - | Yes | No | No | No | Yes | Yes | No | No | - | No | - |
| STOPP C6 | STOPP C6. Antiplatelet agents with vitamin K antagonist, direct thrombin inhibitor or factor Xa inhibitors in patients with stable coronary, cerebrovascular or peripheral arterial disease (No added benefit from dual therapy). | Not Done | Diffcult to exclude case with appropriate anticoagulant/antiplatelet combination (coronary stent less than 12 months) because no information about "coronary stent insertion date" in the database | - | - | - | - | - | - | - | - | - | - | - |
| STOPP C7 | STOPP C7. Ticlopidine in any circumstances (clopidogrel and prasugrel have similar efficacy, stronger evidence and fewer side-effects). | Done | - | Yes | No | No | No | No | No | - | - | - | Yes with EU-PIM | Ticlopidine (B01AC05) |
| STOPP C8 | STOPP C8. Vitamin K antagonist, direct thrombin inhibitor or factor Xa inhibitors for first deep venous thrombosis without continuing provoking risk factors (e.g. thrombophilia) for > 6 months, (no proven added benefit). | Not Done | No information about "thombophilia exploration" in the database | - | - | - | - | - | - | - | - | - | - | - |
| STOPP C9 | STOPP C9. Vitamin K antagonist, direct thrombin inhibitor or factor Xa inhibitors for first pulmonary embolus without continuing provoking risk factors (e.g. thrombophilia) for > 12 months (no proven added benefit). | Not Done | No information about "thombophilia exploration" in the database | - | - | - | - | - | - | - | - | - | - | - |
| STOPP C10 | STOPP C10. NSAID and vitamin K antagonist, direct thrombin inhibitor or factor Xa inhibitors in combination (risk of major gastrointestinal bleeding). | Done | - | Yes | No | No | No | Yes | No | - | - | - | No | - |

| Criteria | Explanations | Programmed criteria (Done/Not Done) | Reasons for nonprogramming | Medication information needed (ATC 5) (Yes/No) | Dosage information needed (Yes/No) | Duration information needed (Yes/No) | Route information needed (Yes/No) | Association of drugs information needed (Yes/No) | Comorbidity information needed (text) (Yes/No) | Association of comorbidities information needed (Yes/No) | Others clinical measures (Yes/No) | Others clinical measures (details) | Strict redundancy between criteria | Strict redundancy between criteria |
|---|---|---|---|---|---|---|---|---|---|---|---|---|---|---|
| STOPP C11 | STOPP C11. NSAID with concurrent antiplatelet agent(s) without PPI prophylaxis (increased risk of peptic ulcer disease) | Done | - | Yes | No | No | No | Yes | No | - | - | - | No | - |
| STOPP D1 | STOPP D1. TriCyclic Antidepressants (TCAs) with dementia, narrow angle glaucoma, cardiac conduction abnormalities, prostatism, or prior history of urinary retention (risk of worsening these conditions). | Done | - | Yes | No | No | No | No | Yes | No | No | - | No | - |
| STOPP D2 | STOPP D2. Initiation of TriCyclic Antidepressants (TCAs) as first-line antidepressant treatment (higher risk of adverse drug reactions with TCAs than with SSRIs or SNRIs). | Not Done | No information about "first-line treatment" in the database | - | - | - | - | - | - | - | - | - | - | - |
| STOPP D3 | STOPP D3. Neuroleptics with moderate-marked antimuscarinic/anticholinergic effects (chlorpromazine, clozapine, flupenthixol, fluphenzine, pipothiazine, promazine, zuclopenthixol) with a history of prostatism or previous urinary retention (high risk of urinary retention). | Done | - | Yes | No | No | No | No | Yes | No | No | - | No | - |
| STOPP D4 | STOPP D4. Selective serotonin re-uptake inhibitors (SSRI's) with current or recent significant hyponatraemia i.e. serum Na+ < 130 mmol/l (risk of exacerbating or precipitating hyponatraemia). | Not Done | No information about blood sodium levels and "current or recent significant hyponatremia" in the database | - | - | - | - | - | - | - | - | - | - | - |
| STOPP D5 | STOPP D5. Benzodiazepines for ≥ 4 weeks (no indication for longer treatment; risk of prolonged sedation, confusion, impaired balance, falls, road traffic accidents; all benzodiazepines should be withdrawn gradually if taken for more than 4 weeks as there is a risk of causing a benzodiazepine withdrawal syndrome if stopped abruptly). | Done | - | Yes | No | Yes | No | No | No | - | - | - | No | - |
| STOPP D6 | STOPP D6. Antipsychotics (i.e. other than quetiapine or clozapine) in those with parkinsonism or Lewy Body Disease (risk of severe extra-pyramidal symptoms) | Done | - | Yes | No | No | No | No | Yes | No | No | - | No | - |
| STOPP D7 | STOPP D7. Anticholinergics/antimuscarinics to treat extra-pyramidal side-effects of neuroleptic medications (risk of anticholinergic toxicity), | Done | - | Yes | No | No | No | No | Yes | No | No | - | No | - |
| STOPP D8 | STOPP D8. Anticholinergics/antimuscarinics in patients with delirium or dementia (risk of exacerbation of cognitive impairment). | Done | - | Yes | No | No | No | No | Yes | No | No | - | No | - |

| Criteria | Explanations | Programmed criteria (Done/Not Done) | Reasons for nonprogramming | Medication information needed (ATC 5) (Yes/No) | Dosage information needed (Yes/No) | Duration information needed (Yes/No) | Route information needed (Yes/No) | Association of drugs information needed (Yes/No) | Comorbidity information needed (text) (Yes/No) | Association of comorbidities information needed (Yes/No) | Others clinical measures (Yes/No) | Others clinical measures (details) | Strict redundancy between criteria | Strict redundancy between criteria |
|---|---|---|---|---|---|---|---|---|---|---|---|---|---|---|
| STOPP D9 | STOPP D9. Neuroleptic antipsychotic in patients with behavioural and psychological symptoms of dementia (BPSD) unless symptoms are severe and other non-pharmacological treatments have failed (increased risk of stroke). | Not Done | No information about "unless symptoms are severe and other nonpharmacological treatments have failed" in the database | - | - | - | - | - | - | - | - | - | - | - |
| STOPP D10 | STOPP D10. Neuroleptics as hypnotics, unless sleep disorder is due to psychosis or dementia (risk of confusion, hypotension, extra-pyramidal side effects, falls). | Done | - | Yes | No | No | No | No | Yes | No | No | - | No | - |
| STOPP D11 | STOPP D11. Acetylcholinesterase inhibitors with a known history of persistent bradycardia (< 60 beats/min.), heart block or recurrent unexplained syncope or concurrent treatment with drugs that reduce heart rate such as beta-blockers, digoxin, diltiazem, verapamil (risk of cardiac conduction failure, syncope and injury). | Done | - | Yes | No | No | No | Yes | Yes | No | Yes | Heart rate | No | - |
| STOPP D12 | STOPP D12. Phenothiazines as first-line treatment, since safer and more efficacious alternatives exist (phenothiazines are sedative, have significant anti-muscarinic toxicity in older people, with the exception of prochlorperazine for nausea/vomiting/vertigo, chlorpromazine for relief of persistent hiccoughs and levomepromazine as an anti-emetic in palliative care ). | Not Done | No information about "first-line treatment" in the database | - | - | - | - | - | - | - | - | - | - | - |
| STOPP D13 | STOPP D13. Levodopa or dopamine agonists for benign essential tremor (no evidence of efficacy) | Done | - | Yes | No | No | No | No | Yes | No | No | - | No | - |
| STOPP D14 | STOPP D14. First-generation antihistamines (safer, less toxic antihistamines now widely available). | Done | - | Yes | No | No | No | No | No | - | - | - | Yes with EU-PIM | Numerous first-generation antihistamines |
| STOPP E1 | STOPP E1. Digoxin at a long-term dose greater than 125µg/day if eGFR < 30 ml/min/1.73m2 (risk of digoxin toxicity if plasma levels not measured). | Not Done | No information about serum creatinine levels in the database | - | - | - | - | - | - | - | - | - | - | - |
| STOPP E2 | STOPP E2. Direct thrombin inhibitors (e.g. dabigatran) if eGFR < 30 ml/min/1.73m2 (risk of bleeding) | Not Done | No information about serum creatinine levels in the database | - | - | - | - | - | - | - | - | - | - | - |
| STOPP E3 | STOPP E3. Factor Xa inhibitors (e.g. rivaroxaban, apixaban) if eGFR < 15 ml/min/1.73m2 (risk of bleeding) | Not Done | No information about serum creatinine levels in the database | - | - | - | - | - | - | - | - | - | - | - |
| STOPP E4 | STOPP E4. NSAID's if eGFR < 50 ml/min/1.73m2 (risk of deterioration in renal function). | Not Done | No information about serum creatinine levels in the database | - | - | - | - | - | - | - | - | - | - | - |

| Criteria | Explanations | Programmed criteria (Done/Not Done) | Reasons for nonprogramming | Medication information needed (ATC 5) (Yes/No) | Dosage information needed (Yes/No) | Duration information needed (Yes/No) | Route information needed (Yes/No) | Association of drugs information needed (Yes/No) | Comorbidity information needed (text) (Yes/No) | Association of comorbidities information needed (Yes/No) | Others clinical measures (Yes/No) | Others clinical measures (details) | Strict redundancy between criteria | Strict redundancy between criteria |
|---|---|---|---|---|---|---|---|---|---|---|---|---|---|---|
| STOPP E5 | STOPP E5. Colchicine if eGFR < 10 ml/min/1.73m2 (risk of colchicine toxicity) | Not Done | No information about serum creatinine levels in the database | - | - | - | - | - | - | - | - | - | - | - |
| STOPP E6 | STOPP E6. Metformin if eGFR < 30 ml/min/1.73m2 (risk of lactic acidosis). | Not Done | No information about serum creatinine levels in the database | - | - | - | - | - | - | - | - | - | - | - |
| STOPP F1 | STOPP F1. Prochlorperazine or metoclopramide with Parkinsonism (risk of exacerbating Parkinsonian symptoms). | Done | - | Yes | No | No | No | No | Yes | No | No | - | No | - |
| STOPP F2 | STOPP F2. PPI for uncomplicated peptic ulcer disease or erosive peptic oesophagitis at full therapeutic dosage for > 8 weeks (dose reduction or earlier discontinuation indicated). | Not Done | No information about peptic ulcer disease severity in the database | - | - | - | - | - | - | - | - | - | - | - |
| STOPP F3 | STOPP F3. Drugs likely to cause constipation (e.g. antimuscarinic/anticholinergic drugs, oral iron, opioids, verapamil, aluminium antacids) in patients with chronic constipation where non-constipating alternatives are available (risk of exacerbation of constipation). | Done | - | Yes | No | No | No | No | Yes | No | No | - | No | - |
| STOPP F4 | STOPP F4. Oral elemental iron doses greater than 200 mg daily (e.g. ferrous fumarate> 600 mg/day, ferrous sulphate > 600 mg/day, ferrous gluconate> 1800 mg/day; no evidence of enhanced iron absorption above these doses). | Done | - | Yes | Yes | No | Yes | No | No | - | - | - | Yes with EU-PIM | Iron supplements (B03AA) |
| STOPP G1 | STOPP G1. Theophylline as monotherapy for COPD (safer, more effective alternative; risk of adverse effects due to narrow therapeutic index). | Done | - | Yes | No | No | No | Yes | Yes | No | No | - | No | - |
| STOPP G2 | STOPP G2. Systemic corticosteroids instead of inhaled corticosteroids for maintenance therapy in moderate-severe COPD (unnecessary exposure to long-term side-effects of systemic corticosteroids and effective inhaled therapies are available). | Not Done | Lack of comprehensive clinical information for which systemic corticosteroids can be prescribed | - | - | - | - | - | - | - | - | - | - | - |
| STOPP G3 | STOPP G3. Anti-muscarinic bronchodilators (e.g. ipratropium, tiotropium) with a history of narrow angle glaucoma (may exacerbate glaucoma) or bladder outflow obstruction (may cause urinary retention). | Done | - | Yes | No | No | No | No | Yes | No | No | - | No | - |
| STOPP G4 | STOPP G4. Benzodiazepines with acute or chronic respiratory failure i.e. pO2 < 8.0 kPa ± pCO2 > 6.5 kPa (risk of exacerbation of respiratory failure). | Done | - | Yes | No | No | No | No | Yes | No | No | - | No | - |

| Criteria | Explanations | Programmed criteria (Done/Not Done) | Reasons for nonprogramming | Medication information needed (ATC 5) (Yes/No) | Dosage information needed (Yes/No) | Duration information needed (Yes/No) | Route information needed (Yes/No) | Association of drugs information needed (Yes/No) | Comorbidity information needed (text) (Yes/No) | Association of comorbidities information needed (Yes/No) | Others clinical measures (Yes/No) | Others clinical measures (details) | Strict redundancy between criteria | Strict redundancy between criteria |
|---|---|---|---|---|---|---|---|---|---|---|---|---|---|---|
| STOPP H1 | STOPP H1. Non-steroidal anti-inflammatory drug (NSAID) other than COX-2 selective agents with history of peptic ulcer disease or gastrointestinal bleeding, unless with concurrent PPI or H2 antagonist (risk of peptic ulcer relapse). | Done | - | Yes | No | No | No | No | Yes | No | No | - | No | - |
| STOPP H2 | STOPP H2. NSAID with severe hypertension (risk of exacerbation of hypertension) or severe heart failure (risk of exacerbation of heart failure). | Done | - | Yes | No | No | No | No | Yes | No | Yes | Systolic and diastolic blood pressure | No | - |
| STOPP H3 | STOPP H3. Long-term use of NSAID (>3 months) for symptom relief of osteoarthritis pain where paracetamol has not been tried (simple analgesics preferable and usually as effective for pain relief) | Not Done | No information about "situation where acetaminophen has not been tried" in the database | - | - | - | - | - | - | - | - | - | - | - |
| STOPP H4 | STOPP H4. Long-term corticosteroids (>3 months) as monotherapy for rheumatoid arthrtitis (risk of systemic corticosteroid side-effects). | Not Done | Lack of comprehensive clinical information for which systemic corticosteroids can be prescribed | - | - | - | - | - | - | - | - | - | - | - |
| STOPP H5 | STOPP H5. Corticosteroids (other than periodic intra-articular injections for mono-articular pain) for osteoarthritis (risk of systemic corticosteroid side-effects). | Not Done | Lack of comprehensive clinical information for which systemic corticosteroids can be prescribed | - | - | - | - | - | - | - | - | - | - | - |
| STOPP H6 | STOPP H6. Long-term NSAID or colchicine (>3 months) for chronic treatment of gout where there is no contraindication to a xanthine-oxidase inhibitor (e.g. allopurinol, febuxostat) (xanthine-oxidase inhibitors are first choice prophylactic drugs in gout). | Done | - | Yes | No | No | No | Yes | Yes | No | No | - | No | - |
| STOPP H7 | STOPP H7. COX-2 selective NSAIDs with concurrent cardiovascular disease (increased risk of myocardial infarction and stroke) | Not Done | Lack of clinical information to identify all patients with concurrent cardiovascular disease in the database | - | - | - | - | - | - | - | - | - | - | - |
| STOPP H8 | STOPP H8. NSAID with concurrent corticosteroids without PPI prophylaxis (increased risk of peptic ulcer disease) | Done | - | Yes | No | No | No | Yes | No | - | - | - | No | - |
| STOPP H9 | STOPP H9. Oral bisphosphonates in patients with a current or recent history of upper gastrointestinal disease i.e. dysphagia, oesophagitis, gastritis, duodenitis, or peptic ulcer disease, or upper gastrointestinal bleeding (risk of relapse/exacerbation of oesophagitis, oesophageal ulcer, oesophageal stricture) | Done | - | Yes | No | No | Yes | No | Yes | No | No | - | No | - |

| Criteria | Explanations | Programmed criteria (Done/Not Done) | Reasons for nonprogramming | Medication information needed (ATC 5) (Yes/No) | Dosage information needed (Yes/No) | Duration information needed (Yes/No) | Route information needed (Yes/No) | Association of drugs information needed (Yes/No) | Comorbidity information needed (text) (Yes/No) | Association of comorbidities information needed (Yes/No) | Others clinical measures (Yes/No) | Others clinical measures (details) | Strict redundancy between criteria | Strict redundancy between criteria |
|---|---|---|---|---|---|---|---|---|---|---|---|---|---|---|
| STOPP I1 | STOPP I1. Antimuscarinic drugs with dementia, or chronic cognitive impairment (risk of increased confusion, agitation) or narrow-angle glaucoma (risk of acute exacerbation of glaucoma), or chronic prostatism (risk of urinary retention). | Done | - | Yes | No | No | No | No | Yes | Yes | No | - | No | - |
| STOPP I2 | STOPP I2. Selective alpha-1 selective alpha blockers in those with symptomatic orthostatic hypotension or micturition syncope (risk of precipitating recurrent syncope) | Done | - | Yes | No | No | No | No | Yes | No | No | - | No | - |
| STOPP J1 | STOPP J1. Sulphonylureas with a long duration of action (e.g. glibenclamide, chlorpropamide, glimepiride) with type 2 diabetes mellitus (risk of prolonged hypoglycaemia). | Done | - | Yes | No | No | No | No | No | - | - | - | Yes with EU-PIM | Numerous sulphonylureas |
| STOPP J2 | STOPP J2. Thiazolidenediones (e.g. rosiglitazone, pioglitazone) in patients with heart failure (risk of exacerbation of heart failure) | Done | - | Yes | No | No | No | No | Yes | No | No | - | No | - |
| STOPP J3 | STOPP J3. Beta-blockers in diabetes mellitus with frequent hypoglycaemic episodes (risk of suppressing hypoglycaemic symptoms). | Not Done | No information about "hypoglycemic episodes" frequence in the database | - | - | - | - | - | - | - | - | - | - | - |
| STOPP J4 | STOPP J4. Oestrogens with a history of breast cancer or venous thromboembolism (increased risk of recurrence). | Done | - | Yes | No | No | No | No | Yes | No | No | - | No | - |
| STOPP J5 | STOPP J5. Oral oestrogens without progestogen in patients with intact uterus (risk of endometrial cancer). | Done | - | Yes | No | No | Yes | Yes | Yes | No | No | - | No | - |
| STOPP J6 | STOPP J6. Androgens (male sex hormones) in the absence of primary or secondary hypogonadism (risk of androgen toxicity; no proven benefit outside of the hypogonadism indication). | Done | - | - | - | - | - | - | - | - | - | - | - | - |
| STOPP K1 | STOPP K1. Benzodiazepines (sedative, may cause reduced sensorium, impair balance). | Not Done | Criterion too broad | - | - | - | - | - | - | - | - | - | - | - |
| STOPP K2 | STOPP K2. Neuroleptic drugs (may cause gait dyspraxia, Parkinsonism). | Not Done | Criterion too broad | - | - | - | - | - | - | - | - | - | - | - |
| STOPP K3 | STOPP K3. Vasodilator drugs (e.g. alpha-1 receptor blockers, calcium channel blockers, long-acting nitrates, ACE inhibitors, angiotensin I receptor blockers, ) with persistent postural hypotension i.e. recurrent drop in systolic blood pressure ≥ 20mmHg (risk of syncope, falls). | Done | - | Yes | No | No | Yes | No | Yes | No | Yes | Systolic and diastolic blood pressure, before and after standing | No | - |

| Criteria | Explanations | Programmed criteria (Done/Not Done) | Reasons for nonprogramming | Medication information needed (ATC 5) (Yes/No) | Dosage information needed (Yes/No) | Duration information needed (Yes/No) | Route information needed (Yes/No) | Association of drugs information needed (Yes/No) | Comorbidity information needed (text) (Yes/No) | Association of comorbidities information needed (Yes/No) | Others clinical measures (Yes/No) | Others clinical measures (details) | Strict redundancy between criteria | Strict redundancy between criteria |
|---|---|---|---|---|---|---|---|---|---|---|---|---|---|---|
| STOPP K4 | STOPP K4. Hypnotic Z-drugs e.g. zopiclone, zolpidem, zaleplon (may cause protracted daytime sedation, ataxia). | Not Done | Criterion too broad | - | - | - | - | - | - | - | - | - | - | - |
| STOPP L1 | STOPP L1. Use of oral or transdermal strong opioids (morphine, oxycodone, fentanyl, buprenorphine, diamorphine, methadone, tramadol, pethidine, pentazocine) as first line therapy for mild pain (WHO analgesic ladder not observed). | Not Done | No information about "first-line therapy" and pain monitoring in the database | - | - | - | - | - | - | - | - | - | - | - |
| STOPP L2 | STOPP L2. Use of regular (as distinct from PRN) opioids without concomitant laxative (risk of severe constipation). | Not Done | No information about "regular use" and pain monitoring in the database | - | - | - | - | - | - | - | - | - | - | - |
| STOPP L3 | STOPP L3. Long-acting opioids without short-acting opioids for break-through pain (risk of persistence of severe pain) | Not Done | No information about pain monitoring in the database | - | - | - | - | - | - | - | - | - | - | - |
| STOPP N1 | STOPP N1. Concomitant use of two or more drugs with antimuscarinic/anticholinergic properties (e.g. bladder antispasmodics, intestinal antispasmodics, tricyclic antidepressants, first generation antihistamines) (risk of increased antimuscarinic/anticholinergic toxicity) | Done | - | Yes | No | No | No | No | No | - | - | - | No | - |
| START A1 | START A1. Vitamin K antagonists or direct thrombin inhibitors or factor Xa inhibitors in the presence of chronic atrial fibrillation. | Done | - | Yes | No | No | No | No | Yes | No | No | - | No | - |
| START A2 | START A2. Aspirin (75 mg – 160 mg once daily) in the presence of chronic atrial fibrillation, where Vitamin K antagonists or direct thrombin inhibitors or factor Xa inhibitors are contraindicated. | Not Done | No information about anticoagulant contraindication in the database | - | - | - | - | - | - | - | - | - | - | - |
| START A3 | START A3. Antiplatelet therapy (aspirin or clopidogrel or prasugrel or ticagrelor) with a documented history of coronary, cerebral or peripheral vascular disease. | Done | - | Yes | No | No | No | No | Yes | No | No | - | No | - |
| START A4 | START A4. Antihypertensive therapy where systolic blood pressure consistently > 160 mmHg and/or diastolic blood pressure consistently >90 mmHg; if systolic blood pressure > 140 mmHg and /or diastolic blood pressure > 90 mmHg, if diabetic. | Done | - | Yes | No | No | No | No | Yes | No | Yes | Systolic and diastolic blood pressure | No | - |
| START A5 | START A5. Statin therapy with a documented history of coronary, cerebral or peripheral vascular disease, unless the patient’s status is end-of-life or age is > 85 years. | Done | - | Yes | No | No | No | No | Yes | No | No | - | No | - |
| START A6 | START A6. Angiotensin Converting Enzyme (ACE) inhibitor with systolic heart failure and/or documented coronary artery disease. | Done | - | Yes | No | No | No | No | Yes | No | No | - | No | - |

| Criteria | Explanations | Programmed criteria (Done/Not Done) | Reasons for nonprogramming | Medication information needed (ATC 5) (Yes/No) | Dosage information needed (Yes/No) | Duration information needed (Yes/No) | Route information needed (Yes/No) | Association of drugs information needed (Yes/No) | Comorbidity information needed (text) (Yes/No) | Association of comorbidities information needed (Yes/No) | Others clinical measures (Yes/No) | Others clinical measures (details) | Strict redundancy between criteria | Strict redundancy between criteria |
|---|---|---|---|---|---|---|---|---|---|---|---|---|---|---|
| START A7 | START A7. Beta-blocker with ischaemic heart disease. | Done | - | Yes | No | No | No | No | Yes | No | No | - | No | - |
| START A8 | START A8. Appropriate beta-blocker (bisoprolol, nebivolol, metoprolol or carvedilol) with stable systolic heart failure. | Done | - | Yes | No | No | No | No | Yes | No | No | - | No | - |
| START B1 | START B1. Regular inhaled b2 agonist or antimuscarinic bronchodilator (e.g. ipratropium, tiotropium) for mild to moderate asthma or COPD. | Done | - | Yes | No | No | No | No | Yes | No | No | - | No | - |
| START B2 | START B2. Regular inhaled corticosteroid for moderate-severe asthma or COPD, where FEV1 <50% of predicted value and repeated exacerbations requiring treatment with oral corticosteroids. | Not Done | No information about "repeated exacerbations requiring treatment with oral corticosteroids" and FEV1 in the database | - | - | - | - | - | - | - | - | - | - | - |
| START B3 | START B3. Home continuous oxygen with documented chronic hypoxaemia (i.e. pO2 < 8.0 kPa or 60 mmHg or SaO2 < 89%) | Not Done | No information about pO2 and SaO2 in the database | - | - | - | - | - | - | - | - | - | - | - |
| START C1 | START C1. L-DOPA or a dopamine agonist in idiopathic Parkinson's disease with functional impairment and resultant disability. | Done | - | Yes | No | No | No | No | Yes | No | No | - | No | - |
| START C2 | START C2. Non-TCA antidepressant drug in the presence of persistent major depressive symptoms. | Done | - | Yes | No | No | No | No | Yes | No | Yes | Geriatric Depressio n Scale (GDS) | No | - |
| START C3 | START C3. Acetylcholinesterase inhibitor (e.g. donepezil, rivastigmine, galantamine) for mild-moderate Alzheimer's dementia or Lewy Body dementia (rivastigmine). | Not Done | Acetylcholinesterase inhibitor are not recommended according to HAS and not reimbursed by French social security | - | - | - | - | - | - | - | - | - | - | - |
| START C4 | START C4. Topical prostaglandin, prostamide or beta-blocker for primary open-angle glaucoma. | Done | - | Yes | No | No | No | No | Yes | No | No | - | No | - |
| START C5 | START C5. Selective serotonin reuptake inhibitor (or SNRI or pregabalin if SSRI contraindicated) for persistent severe anxiety that interferes with independent functioning. | Not Done | No information about "persistent severe anxiety that interferes with independent functioning" in the database | - | - | - | - | - | - | - | - | - | - | - |
| START C6 | START C6. Dopamine agonist (ropinirole or pramipexole or rotigotine) for Restless Legs Syndrome, once iron deficiency and severe renal failure have been excluded. | Not Done | No information about " iron deficiency" and "renal failure" severity in the database | - | - | - | - | - | - | - | - | - | - | - |
| START D1 | START D1. Proton Pump Inhibitor with severe gastro-oesophageal reflux disease or peptic stricture requiring dilatation. | Done | - | Yes | No | No | No | No | Yes | No | No | - | No | - |

| Criteria | Explanations | Programmed criteria (Done/Not Done) | Reasons for nonprogramming | Medication information needed (ATC 5) (Yes/No) | Dosage information needed (Yes/No) | Duration information needed (Yes/No) | Route information needed (Yes/No) | Association of drugs information needed (Yes/No) | Comorbidity information needed (text) (Yes/No) | Association of comorbidities information needed (Yes/No) | Others clinical measures (Yes/No) | Others clinical measures (details) | Strict redundancy between criteria | Strict redundancy between criteria |
|---|---|---|---|---|---|---|---|---|---|---|---|---|---|---|
| START D2 | START D2. Fibre supplements (e.g. bran, ispaghula, methylcellulose, sterculia) for diverticulosis with a history of constipation. | Not Done | No exhaustive information about "fiber supplements" in the drug database because some fiber supplements are considered food supplements, and not drugs | - | - | - | - | - | - | - | - | - | - | - |
| START E1 | START E1. Disease-modifying anti-rheumatic drug (DMARD) with active, disabling rheumatoid disease. | Done | - | Yes | No | No | No | No | Yes | No | No | - | No | - |
| START E2 | START E2. Bisphosphonates and vitamin D and calcium in patients taking long-term systemic corticosteroid therapy. | Done | - | Yes | No | No | No | Yes | No | - | - | - | No | - |
| START E3 | START E3. Vitamin D and calcium supplement in patients with known osteoporosis and/or previous fragility fracture(s) and/or (Bone Mineral Density T-scores more than -2.5 in multiple sites). | Done | - | Yes | No | No | No | Yes | Yes | No | No | - | No | - |
| START E4 | START E4. Bone anti-resorptive or anabolic therapy (e.g. bisphosphonate, strontium ranelate, teriparatide, denosumab) in patients with documented osteoporosis, where no pharmacological or clinical status contraindication exists (Bone Mineral Density T-scores -> 2.5 in multiple sites) and/or previous history of fragility fracture(s). | Done | - | Yes | No | No | No | No | Yes | No | No | - | No | - |
| START E5 | START E5. Vitamin D supplement in older people who are housebound or experiencing falls or with osteopenia (Bone Mineral Density T-score is > -1.0 but < -2.5 in multiple sites). | Done | - | Yes | No | No | No | No | Yes | No | No | - | No | - |
| START E6 | START E6. Xanthine-oxidase inhibitors (e.g. allopurinol, febuxostat) with a history of recurrent episodes of gout. | Done | - | Yes | No | No | No | No | Yes | No | No | - | No | - |
| START E7 | START E7. Folic acid supplement in patients taking methotexate. | Done | - | Yes | No | No | No | Yes | No | - | - | - | No | - |
| START F1 | START F1. ACE inhibitor or Angiotensin Receptor Blocker (if intolerant of ACE inhibitor) in diabetes with evidence of renal disease i.e. dipstick proteinuria or microalbuminuria (>30mg/24 hours) with or without serum biochemical renal impairment. | Done | - | Yes | No | No | No | No | Yes | Yes | No | - | No | - |

| Criteria | Explanations | Programmed criteria (Done/Not Done) | Reasons for nonprogramming | Medication information needed (ATC 5) (Yes/No) | Dosage information needed (Yes/No) | Duration information needed (Yes/No) | Route information needed (Yes/No) | Association of drugs information needed (Yes/No) | Comorbidity information needed (text) (Yes/No) | Association of comorbidities information needed (Yes/No) | Others clinical measures (Yes/No) | Others clinical measures (details) | Strict redundancy between criteria | Strict redundancy between criteria |
|---|---|---|---|---|---|---|---|---|---|---|---|---|---|---|
| START G1 | START G1. Alpha-1 receptor blocker with symptomatic prostatism, where prostatectomy is not considered necessary. | Done | - | Yes | No | No | No | No | Yes | No | No | - | Yes with START_G2 | Monotherapy with 5-alpha reductase inhibitor can be appropriate |
| START G2 | START G2. 5-alpha reductase inhibitor with symptomatic prostatism, where prostatectomy is not considered necessary. | Done | - | Yes | No | No | No | No | Yes | No | No | - | Yes with START_G1 | Monotherapy with alpha-1 receptor blocker can be appropriate |
| START G3 | START G3. Topical vaginal oestrogen or vaginal oestrogen pessary for symptomatic atrophic vaginitis. | Done | - | Yes | No | No | Yes | No | Yes | No | No | - | No | - |
| START H1 | START H1. High-potency opioids in moderate-severe pain, where paracetamol, NSAIDs or low-potency opioids are not appropriate to the pain severity or have been ineffective. | Not Done | No information about "paracetamol, NSAIDs or low-potency opioids are not appropriate to the pain severity or have been ineffective" and pain monitoring in the database | - | - | - | - | - | - | - | - | - | - | - |
| START H2 | START H2. Laxatives in patients receiving opioids regularly. | Done | - | Yes | No | No | No | Yes | No | - | - | - | No | - |
| START I1 | START I1. Seasonal trivalent influenza vaccine annually | Not Done | No exhaustive information about vaccination in the database | - | - | - | - | - | - | - | - | - | - | - |
| START I2 | START I2. Pneumococcal vaccine at least once after age 65 according to national guidelines | Not Done | No comprehensive information on past immunization history in the database | - | - | - | - | - | - | - | - | - | - | - |
